# Supplementary material for: The Firefighter problem with dynamic defence costs
Source: PLoS One. 2026 Feb 6;21(2):e0341222. doi: 10.1371/journal.pone.0341222 (PMC12880756; doi:10.1371/journal.pone.0341222)
Supplement: Attachment — Submitted filename: PONE-D-25-17298.pdf [file pone.0341222.s013.pdf]

Report on

PONE-D-25-17298

Title: The Firefighter problem with dynamic defence costs

This paper is based on the firefighter problem with dynamic defence costs. In this paper authors have studied The Firefighter Problem that is a single-player game modelling the spread of contagion (e.g. rumours, diseases) on a graph, where the player's objective is to defend vertices to protect at least a given number. Through a variant (The Cost Function) for the Firefighter Problem, it is shown that in monadic second-order logic, fixed-parameter is tractable with respect to treewidth, budget, and maximum time step. To complement theoretical findings, empirical investigation are considered to compare performance of cost, threat and degree-based heuristics under various cost functions. Relative effectiveness of heuristics, depends heavily on the structure of graph that are used in the process, whereas degree-based heuristics tend are considered to perform worse than state-based strategies.

The authors have mentioned Graph theory in the paper but mathematical tools of graph theory are not used in any results neither the paper is scientifically so sound to be considered for the publication in Plos One. I, therefore, do not suggest to accept this paper for its publication in Plos One. However it could be valuable for the specific journal. The authors can consider specialized journal for the publication of this article.
