# Supplementary material for: The Firefighter problem with dynamic defence costs
Source: PLoS One. 2026 Feb 6;21(2):e0341222. doi: 10.1371/journal.pone.0341222 (PMC12880756; doi:10.1371/journal.pone.0341222)
Supplement: Attachment — Submitted filename: plos1.pdf [file pone.0341222.s014.pdf]

Review Report

In the paper entitled **"The Firefighter problem with dynamic defence costs"**, the authors have introduced a variant of the Firefighter problem called The Cost Function Firefighter Problem, in which each vertex has a cost to defend that varies with time and the burning state of the graph. To complement theoretical findings, they compare performance of cost, threat and degree-based heuristics under various cost functions. Their findings explain that the relative effectiveness of these heuristics depends heavily on the structure of graph used in the process, but degree-based heuristics tend to perform worse than state-based strategies. This manuscript has been presented at an international conference, 2024.
I suggest to **accept the manuscript**, after authors address the following;

1. How well do these models capture real-world dynamics like delay, uncertainty, or feedback loops?
2. Has the problem been simulated on real networks (e.g., social, transportation, biological)?
3. Can the problem be extended to weighted or directed graphs, and how does that affect solution strategies?
4. What algorithms are commonly used to solve or approximate solutions to the Firefighter Problem?
5. What are the best-known approximation ratios for specific versions of the problem?
6. On which graph classes is the Firefighter Problem solvable in polynomial time?
7. What are the known variants of the Firefighter Problem?
8. What are the limitations of this study?
9. Aligns text to both margins evenly.
10. Figure 8, 9, 10, 11 and 12 are not clear.
11. Make sure that all references are cited within the manuscript.
