# Supplementary material for: The Firefighter problem with dynamic defence costs
Source: PLoS One. 2026 Feb 6;21(2):e0341222. doi: 10.1371/journal.pone.0341222 (PMC12880756; doi:10.1371/journal.pone.0341222)
Supplement: Attachment — Submitted filename: Review Report-for Plos.docx [file pone.0341222.s015.docx]

**Manuscript ID: PONE-D-25-17298**

**Title: *The Firefighter Problem with Dynamic Defence Costs***

**Strengths**

- Excellent structure and clarity throughout.
- Strong theoretical foundation and clear NP-hardness reductions.
- Effective use of MSO logic for parameterized complexity.
- Experimental evaluation is thorough and includes both random and real-world graphs.

**Minor Issues & Suggested Revisions**

**1. Abstract Improvements**

- Line: “This problem is computationally hard, but can be solved efficiently...”
  - *Suggestion:* Add a brief example or graph class for which it is efficiently solvable to make the sentence self-contained.
- Line: “We introduce a variant...”
  - *Suggestion:* Consider emphasizing the novelty more clearly — e.g., highlight that it is the first to incorporate time-dependent vertex defence costs.

**2. Notation and Definitions**

- **Definition 2 (Strategy)**
  - The multi-index notation (e.g., djid_{ji}) is clear, but it may help to include a small illustrative figure for visual clarity.
- **Definition 3 (State Mapping)**
  - It would be beneficial to provide a visual timeline or state diagram for the example.

**3. Typographical & Language Refinements**

- **Throughout:** Minor grammar enhancements can improve flow:
  - “Let the target k=n+3mk = n + 3m, the budget b=2b = 2 and over the set of times…” *Change to:* “Let the target be k=n+3mk = n + 3m, with a budget b=2b = 2, over the time steps…”
- **Page 2, Line 23:** “We have designed Cost-Fire to model population heterogeneity...”
  - *Suggestion:* Replace “have designed” with “designed” for consistency in tense.
- **Page 6, Example 5:** Consider formatting the piecewise function using LaTeX-style brackets or clearer indentation.

**4. Table Formatting**

- **Table 1:** Summary is excellent, but could use clearer column separation and improved caption wording:
  - Caption Suggestion: “Summary of tractability and hardness results for *Fire* and *Cost-Fire* across graph classes.”

**5. Figures**

- **Figures 1, 2, 3, 4, etc.**
  - *Suggestion:* Ensure all figures have consistent formatting and high resolution. Captions should explicitly state what is being shown and its relevance to the discussion.

**6. Section 3.6 - PathContainable**

- Excellent theory, but the term “PathContainable” could be better motivated. Consider including a brief paragraph before the definition to provide an intuitive explanation of the motivation and a real-world analogy (e.g., proximity-based decision-making in epidemics).

Cite the latest work.

1. Cai, G., Zheng, X., Gao, W., & Guo, J. (2024). Self-extinction characteristics of fire extinguishing induced by nitrogen injection rescue in an enclosed urban utility tunnel. Case Studies in Thermal Engineering, 59, 104478. doi: https://doi.org/10.1016/j.csite.2024.104478
2. Hao, R., & Yang, X. (2024). Multiple-output quantile regression neural network. Statistics and Computing, 34(2), 89. doi: 10.1007/s11222-024-10408-6
3. Li, L., Xia, Y., Ren, S., & Yang, X. (2025). Homogeneity Pursuit in the Functional-Coefficient Quantile Regression Model for Panel Data with Censored Data, 29(3), 323-348. doi:10.1515/snde-2023-0024
4. Xiong, W., He, D., & Du, H. (2025). Learning economic model predictive control via clustering and kernel-based Lipschitz regression. Journal of the Franklin Institute, 362(12), 107787. doi: https://doi.org/10.1016/j.jfranklin.2025.107787
